# A case of abdominal aortic aneurysm presenting as symptomatic disseminated intravascular coagulation treated with endovascular aneurysm repair and postoperative administration of Nafamostat mesylate

**DOI:** 10.1186/s40792-024-01926-6

**Published:** 2024-05-16

**Authors:** Shinichi Tanaka, Takahiro Ohmine

**Affiliations:** Department of Surgery, Hiroshima Red Cross Hospital & Atomic-Bomb Survivors Hospital, 1-9-6 Sendamachi, Naka-Ku, Hiroshima, 730-8619 Japan

**Keywords:** Disseminated intravascular coagulation (DIC), Abdominal aortic aneurysm (AAA), Endovascular aneurysm repair (EVAR)

## Abstract

**Background:**

Cases of abdominal aortic aneurysm discovered as purpura on the extremities with disseminated intravascular coagulation (DIC) are rare. The number of currently established strategies for the control of DIC with aortic aneurysm is limited.

**Case presentation:**

An 85-year-old woman was referred to the hematology department because of purpura on her shoulder and thigh. Enhanced fibrinolytic-type DIC was diagnosed by a blood test. Enhanced computed tomography (CT) revealed 60-mm abdominal aortic and 42-mm right common iliac aneurysms. We performed endovascular aneurysm repair (EVAR) and coiling of the right internal iliac artery with postoperative administration of Nafamostat mesylate. The patient promptly recovered from DIC, and the purpura gradually disappeared.

**Conclusions:**

We safely performed EVAR with postoperative administration of Nafamostat mesylate for an abdominal aortic aneurysms that presented as symptomatic DIC.

## Background

Aortic aneurysms associated with disseminated intravascular coagulation (DIC) are occasionally encountered, but are rarely symptomatic. DIC associated with aortic aneurysms is of the enhanced fibrinolytic type. Treatment for DIC involves treatment of the underlying disease. However, surgical intervention for DIC associated with aortic aneurysm can lead to fatal bleeding. There are limited established strategies for the management of aneurysm-related DIC, but anticoagulants are one strategy. Here, we report a case of abdominal aortic aneurysm (AAA) and common iliac artery aneurysm (CIAA) with symptomatic DIC that we successfully treated with endovascular aneurysm repair (EVAR) and postoperative administration of Nafamostat mesylate.

## Case presentation

**Fig. 1:**
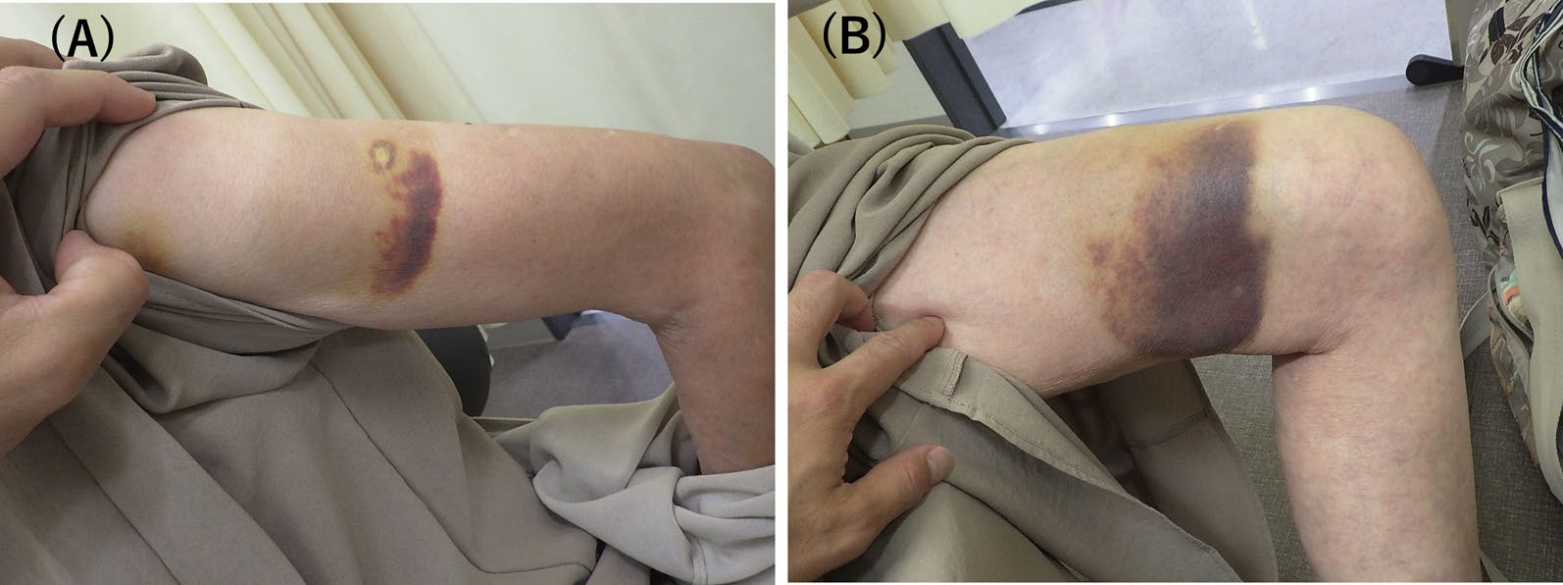
Marked purpura on the upper limb (**A**) and left thigh (**B**) at admission

An 88-year-old woman who presented with upper and lower limb purpura visited the hematology department (Fig. 1A, B). Enhanced fibrinolytic-type DIC was diagnosed by a blood test. The significant laboratory findings (Table 1) were as follows: platelet count, 77,000 cells/mL; a decreased fibrinogen level (128 mg/dL); and an increased fibrin/fibrinogen degradation products level (47.5 μg/mL). The diagnosis of DIC was made according to the diagnostic criteria for DIC established by the Japanese Society on Thrombosis and Hemostasis (4 points).

**Table 1:** Laboratory findings of the patient on admission

Valuables	On admission	Reference range
White blood cell count (/μl)	6400	3300–8700
Hemoglobin (g/dl)	12.5	13.3–16.8
Platelet count (× 103/μl)	77	15.0–35.0
FDP (μg/ml)	47.5	< 5.0
Fibrinogen (mg/dl)	128	200–400
Antithrombin III (%)	99.4	80–130
APTT (s)	29.2	24.0–37.7
PT (s)	12.5	10.1–12.7
D-dimer (μg/ml)	36.5	< 1.0
TAT (ng/ml)	35.8	< 3.9
Serum creatine (mg/dl)	0.62	0.65–1.07
DIC score	4	

*FDP* fibrin/fibrinogen degradation products; *APTT* activated partial thromboplastin time; *PT* prothrombin time; *TAT* thrombin–antithrombin complex; *DIC* disseminated intravascular coagulation

**Fig. 2:**
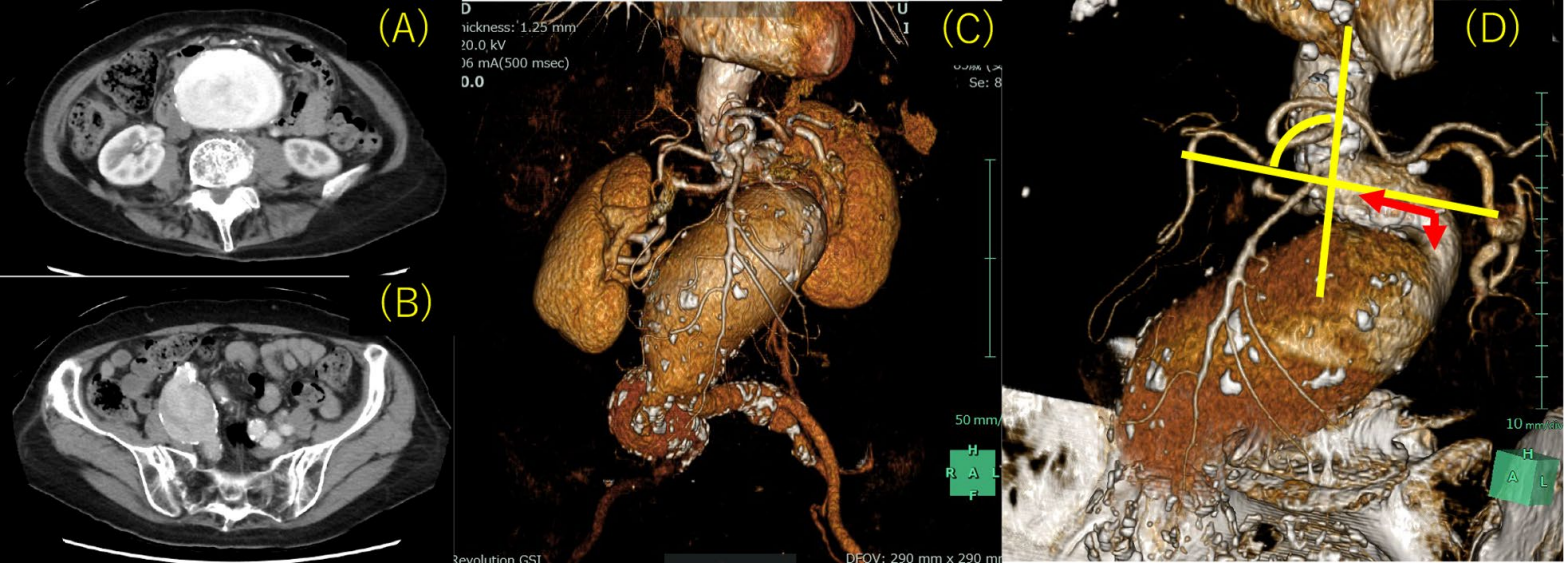
**A** Axial computed tomography images showing an abdominal aortic aneurysm. **B** Axial computed tomography images showing a right common iliac artery aneurysm. **C** Three-dimensional enhanced computed tomography image showing the presence of aneurysms in the abdominal aorta and right common iliac artery. **D** Preoperative volume rendering arteriography revealed that the neck angulation was 89.1 degrees, and the neck length was 17.9 mm

**Fig. 3:**
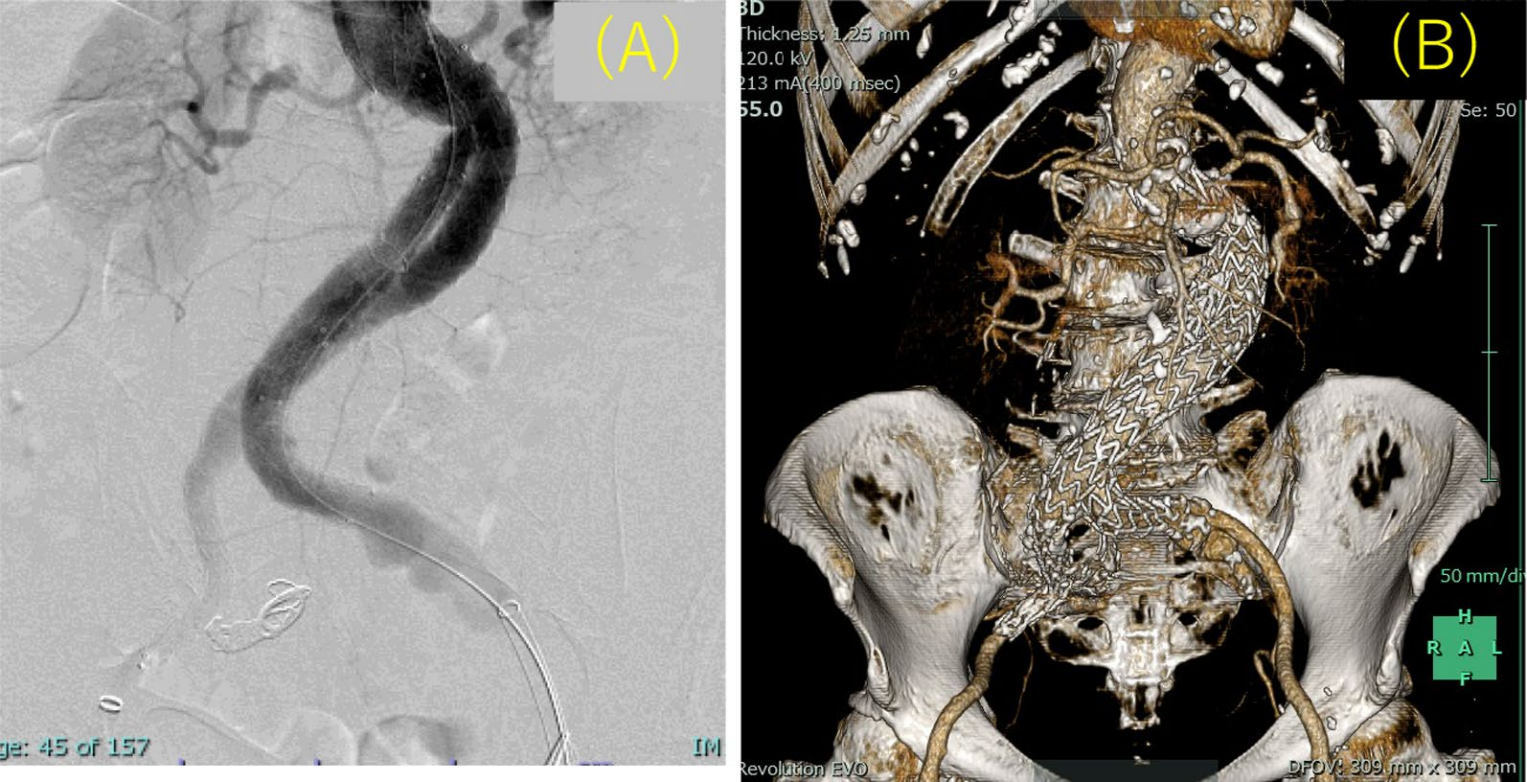
**A** Aortography after stent graft insertion. The abdominal aortic aneurysm and right common iliac artery aneurysm were excluded without any obvious endoleak, and the bilateral renal arteries were preserved. **B** Postoperative three-dimensional computed tomography findings

Contrast-enhanced computed tomography (CT) revealed a 60 mm pararenal AAA, a 42 mm right CIAA, and 19 mm right internal iliac artery dilation (Fig. 2A, B, C). In the absence of other obvious reasons for DIC, we hypothesized that DIC was associated with an AAA and a right CIAA. The angle of the infrarenal neck was severe (89.1°) (Fig. 2D). Although the patient’s anatomical status fell outside the recommended indications for endovascular aneurysm repair (EVAR) and after considering the patient’s advanced age and tendency for bleeding, it was thought that open repair would be excessively invasive; therefore, we chose the endovascular approach. Percutaneous coil embolization of the right internal iliac artery using peripheral HydroCoils (Azur; Terumo, Tokyo, Japan) was carried out prior to EVAR. EVAR was then performed under general anesthesia. An introducer sheath was inserted after the bilateral femoral artery and bilateral brachial artery were cut open. Bilateral renal artery stent placement was attempted to preserve the flow. However, although wiring to the renal artery was successfully performed using a bilateral brachial artery approach, a catheter was not used because of the angle of renal arterial bifurcation. Renal artery stent placement was abandoned. An Endurant II main body stent graft (Medtronic, Santa Rosa, CA, USA) was advanced to the infrarenal artery and inserted into the appropriate location. In the end, a main body stent graft was placed directly below both renal arteries. For the contralateral leg, a stent graft was inserted into the left common iliac artery, and for the ipsilateral leg, a stent graft was inserted into the right external iliac artery. Final digital subtraction angiography demonstrated that the AAA and right CIAA were excluded by the stent graft without visible endoleak, with patency of bilateral renal arteries (Fig. 3A). The intraoperative blood loss was 1456 mL. The patient received 6 units of red blood cells and 10 units of platelet concentrates during the procedure. To prevent thrombus formation associated with sheath implantation, heparin was administered during EVAR, and its effect was reversed with protamine after the procedure.

**Fig. 4:**
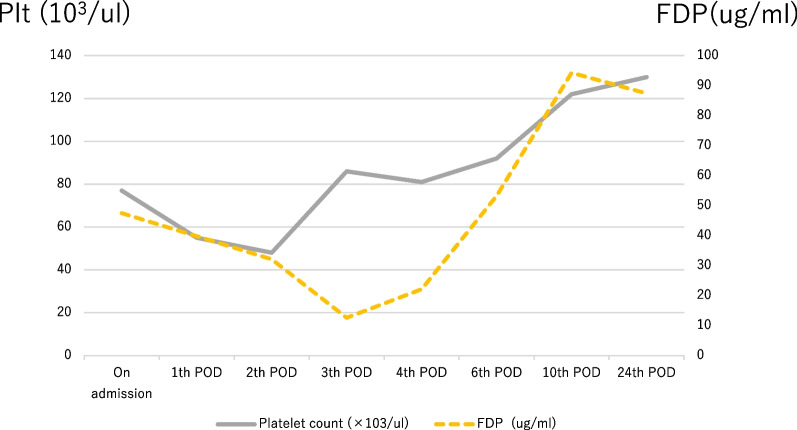
Perioperative treatment course and changes in disseminated intravascular coagulation data. *FDP* fibrin/fibrinogen degradation products, platelet count, *POD* postoperative day

On postoperative day 1, the DIC score worsened to 6 with a drop in platelet count. Nafamostat mesylate was administered intravenously at 200 mg/day. On postoperative day 3, the patient was weaned off DIC with a score of 2, and Nafamostat mesylate was discontinued. The platelet count quickly increased (Fig. 4), and the purpura on the extremities quickly disappeared. The postoperative course was uneventful, and the patient was discharged from the hospital 9 days after surgery. Postoperative CT revealed aneurysm exclusion, no endoleak and patency of the bilateral renal arteries (Fig. 3B). The patient is undergoing outpatient follow-up and is progressing without any bleeding tendency.

## Discussion

Aortic aneurysms associated with DIC are occasionally encountered, but are rarely symptomatic. Only approximately 0.5%–1.0% of aortic aneurysms present a bleeding tendency due to aneurysm-related DIC [1].

Activated coagulation is observed in DIC. However, the degree of fibrinolytic activation depends on the disease type [2]. DIC can be classified as suppressed fibrinolytic, balanced fibrinolytic, or enhanced fibrinolytic [2].

Unlike suppressed fibrinolytic DIC caused by infections such as sepsis, DIC associated with aortic aneurysms characterized by fibrinolysis is markedly enhanced, and bleeding is more common [2].

The preoperative TAT, FDP, and D-dimer values measured in this patient were all elevated (TAT 35.8 ng/ml, FDP 47.5 µg/ml, D-dimer 36.5 µg/ml). In addition, when bleeding symptoms were recognized but when organ symptoms were not, the patient’s FDP was elevated more than D-dimer was. Due to the only one preoperative blood test and lack of PIC measurements, it was not clear if the patient strictly met the criteria for an enhanced fibrinolytic type of fibrosis [2]. However, it was presumed that this patient had DIC that was classified as either the balanced fibrinolytic type or the enhanced fibrinolytic type.

Treatment for DIC consists of treating the underlying disease. However, treatment strategies for AAAs presenting with DIC are unclear, as there is still no high-level evidence [3].

Considering the patient's advanced age and the fact that she and her family requested a less invasive treatment, we proposed EVAR. We decided to go ahead with surgery because her general condition was stable. On postoperative day 1, the DIC score worsened to 6 and it was determined that any medical treatment should be added. Nafamostat mesylate has antiplasmin activity in addition to antithrombin activity and there is less concern about side effects that may contribute to bleeding tendency. Additionally, many reports have described successful treatment in patients with aortic aneurysms [3]. On postoperative day 3, the patient weaned off DIC with a score of 2, and Nafamostat mesylate was discontinued.

There are several reports of the use of recombinant human soluble thrombomodulin as an anticoagulant therapy for controlling DIC with AAA [4, 5]. However, there is little reported experience in administering this drug to DIC patients with underlying diseases other than hematopoietic malignancies, infections, or solid tumors, and its efficacy and safety have not been established for the treatment of aortic aneurysms. The pharmacological mechanism of this drug in treating AAA is also unknown.

In particular, there are a few established options and no clear strategies regarding other medical therapies, such as anticoagulation (unfractionated heparin, heparin, direct oral anticoagulation), replacement therapy (fresh frozen plasma, platelet concentrates) and antifibrinolytic therapy (tranexamic acid), and the optimal timing of treatment for DIC associated with aortic aneurysm is also unknown.

Treatment with EVAR followed by the use of Nafamostat mesylate in the early postoperative period enabled the quick resolution of the patient’s DIC and improvement of the symptomatic bleeding tendency. However, further research is needed on the optimal timing of the use of Nafamostat mesylate, specifically whether this drug should be administered preoperatively or postoperatively.

DIC can be caused by endoleak alone at any time during EVAR or after the procedure. Because the neck from the renal arteries was short and highly curved, there was concern that type I endoleak would occur. Initially, we planned to extend the neck length by placing stent grafts in both renal arteries and then placing the stent grafts. As a result, a stent graft was placed without placing stent grafts in the renal arteries due to the difficulty of the procedure, and fortunately, no type I endoleak was observed. To prevent type I endoleaks, we selected the Endurant stent graft system, which has greater flexibility due to the use of wire-shaped M-shaped stents and suprarenal active fixation by a suprarenal stent.

Inferior mesenteric artery patency (IMA) with an IMA ≥ 3.0 mm and lumbar artery length of ≥ 2.0 mm are reported to be independent risk factors type II endoleak [6]. Preoperative CT images of this patient showed that the IMA was patent but had a diameter of 1 mm, and none of the lumbar arteries were larger than 2 mm; therefore, preoperative coil embolization to prevent type II endoleak was considered unnecessary. Even if there is no problem in the short term, it is necessary to carefully follow up on the presence or absence of endoleak using contrast-enhanced CT during a long-term follow-up.

Our research has certain limitations. Although PIC and α2PI values were missing in this study, they are important markers for analyzing the pathology of DIC in detail. Furthermore, the presence or absence of α2PI deficiency may predict the amount of intraoperative bleeding.

## Conclusions

We report a rare case of pararenal AAA discovered as purpura of the extremities with DIC, which was successfully treated with EVAR and postoperative administration of Nafamostat mesylate.

## Data Availability

Raw data were generated at Hiroshima Red Cross Hospital & Atomic-bomb Survivors Hospital. Derived data supporting the findings of this study are available from the corresponding author on request.

## Abbreviations

DIC: Disseminated intravascular coagulation
CT: Computed tomography
AAA: Abdominal aortic aneurysm
CIAA: Common iliac artery aneurysm
EVAR: Endovascular aneurysm repair
IMA: Inferior mesenteric artery

## References

[1] Levi M, Ten Cate H (1999). Disseminated intravascular coagulation. N Engl J Med.

[2] Yamada S, Asakura H (2021). Management of disseminated intravascular coagulation associated with aortic aneurysm and vascular malformations. Int J Hematol.

[3] Yamada S, Asakura H (2022). Therapeutic strategies for disseminated intravascular coagulation associated with aortic aneurysm. Int J Mol Sci.

[4] Tanigawa Y, Yamada Y, Nakamura K (2021). Preoperative disseminated intravascular coagulation complicated by thoracic aortic aneurysm treated using recombinant human soluble thrombomodulin. Medicine.

[5] Hoshina K, Kaneko M, Hosaka A (2012). Lessons learned from a case of abdominal aortic aneurysm accompanied by unstable coagulopathy. Case Rep Vasc Med.

[6] Samura M, Morikage N, Otsuka R (2020). Endovascular aneurysm repair with inferior mesenteric artery embolization for preventing type II endoleak a prospective randomized controlled trial. Ann Surg.

